# Pathogenic roles of alterations in vitamin D and vitamin D receptor in gastric tumorigenesis

**DOI:** 10.18632/oncotarget.15298

**Published:** 2017-02-11

**Authors:** Chao Du, Shiming Yang, Xiaoyan Zhao, Hui Dong

**Affiliations:** ^1^ Department of Gastroenterology, Xinqiao Hospital, Third Military Medical University, Chongqing, China; ^2^ Department of Gastroenterology and Hepatology, Chengdu Military General Hospital, Chengdu, Sichuan Province, China; ^3^ Division of Gastroenterology, Department of Medicine, School of Medicine, University of California, San Diego, California, USA

**Keywords:** vitamin D, vitamin D receptor, gastric cancer, tumorigenesis

## Abstract

Gastric cancer is currently the second leading cause of cancer-related death worldwide, especially in Japan, Korea and China, and the 5-year survival rate of gastric cancer is less than 30%. Thus, it is important to shed more lights on novel agents to prevent gastric cancer or to improve survival rate of the patients. Vitamin D not only maintains calcium and bone homeostasis, but also mostly inhibits tumor genesis, invasion, and metastasis through activation of vitamin D receptor. Although epidemiological results are not consistent, accumulating evidence from gastric cancer cells, animal models, and clinical trials suggest that vitamin D deficiency may increase the risk and mortality of gastric cancer, but vitamin D supplement might be a safe and economical way to prevent or treat gastric cancer. Here, we reviewed the current studies on vitamin D and its receptor and focused on the pathogenic roles of their alterations in gastric tumorigenesis.

## INTRODUCTION

Gastric cancer is the fourth most common cancer and the second leading cause of cancer-related death in the world. An estimated 1 million new cases of gastric cancer occurred and over 0.7 million patients died worldwide in 2012 only [1]. Currently, gastric cancer is difficult to prevent and cure because of the poor understanding of its pathogenesis and difficulty in its early diagnosis. Even worse, the future burden of gastric cancer is expected to rise with the increase in worldwide population and aging process [2]. Therefore, gastric cancer is regarded as a major public health problem in the world. Multiple therapies (surgery, chemotherapy, radiotherapy, immunotherapy, etc.) are applied to gastric cancer although surgical resection is considered the primary choice for the early stage [3, 4]. Despite of much progress in the pathogenesis, diagnosis and treatment of gastric cancer, its 5-year survival rate is still less than 30% [5]. Therefore, it is urgent to investigate gastric tumorigenesis and to elucidate the underlying molecular mechanisms so that the best ways for preventment and treatment could be developed to decrease the current high morbidity and mortality of gastric cancer.

It is well known that vitamin D plays an important role in maintaining calcium and bone homeostasis and participates in a variety of biological processes in our body as well [6]. It has long been thought low vitamin D status and inadequate calcium intake are important risk factors for various types of human cancer. As early as in 1980, the ultraviolet-B (UVB)-vitamin D-cancer hypothesis was first proposed by Garland [7] who demonstrated that vitamin D is a protective factor against the development of colon cancer. Since then, numerous studies have shown that vitamin D could inhibit the tumorigenesis and prevent tumor progression of breast, colon, skin, pancreas and many other cancers [8–12]. The potential anti-tumor mechanisms of vitamin D may be relevant to its specific receptor, vitamin D receptor (VDR) [13]. VDR is a member of the steroid hormone receptor superfamily of ligand-activated transcription factors [14]. An interaction of vitamin D and VDR can induce a cascade of gene regulation and cell signaling to play important roles in their anti-tumor mechanisms, such as suppression of proliferation, stimulation of apoptosis and autophagy, inhibition of angiogenesis, regulation of immune system and so on [15, 16]. Although several reviews on vitamin D and VDR in some types of human cancer were published, there is no a systematic review on their roles in gastric cancer in the literatures so far. Therefore, in this review, we try to assess the association between vitamin D/VDR and gastric cancer, to explore their multiple anti-tumor mechanisms, and to analyze the safety and validity of vitamin D in the clinical therapy for gastric cancer.

## VITAMIN D: SOURCES, METABOLISM AND RECEPTOR

### Vitamin D sources

Vitamin D is not really a vitamin but a prohormone of the steroid hormone calcitriol, which was first discovered and named by McCollum in 1922 [17]. Although at least ten kinds of vitamin D have been found, the most important forms of vitamin D relevant to human health are vitamin D_2_ (ergocalciferol) and vitamin D_3_ (cholecalciferol). However, vitamin D_2_ and D_3_ are shortage in our normal dietary [18]. Abundant vitamin D is synthesized in the skin when exposed to sunlight [19]. In brief, the UVB (290-315nm) transforms 7-dehydrocholesterol into previtamin D_3_ in the skin, and then previtamin D_3_ is further converted into vitamin D by thermal isomerization [20, 21]. In addition, food supplement, such as normal dietary (the least source of vitamin D), fortified food (egg, milk, salmon, etc.) and concentrated natural food (e.g. cod liver oil), is another subordinate source of vitamin D [22].

### Vitamin D metabolism

**Figure 1:**
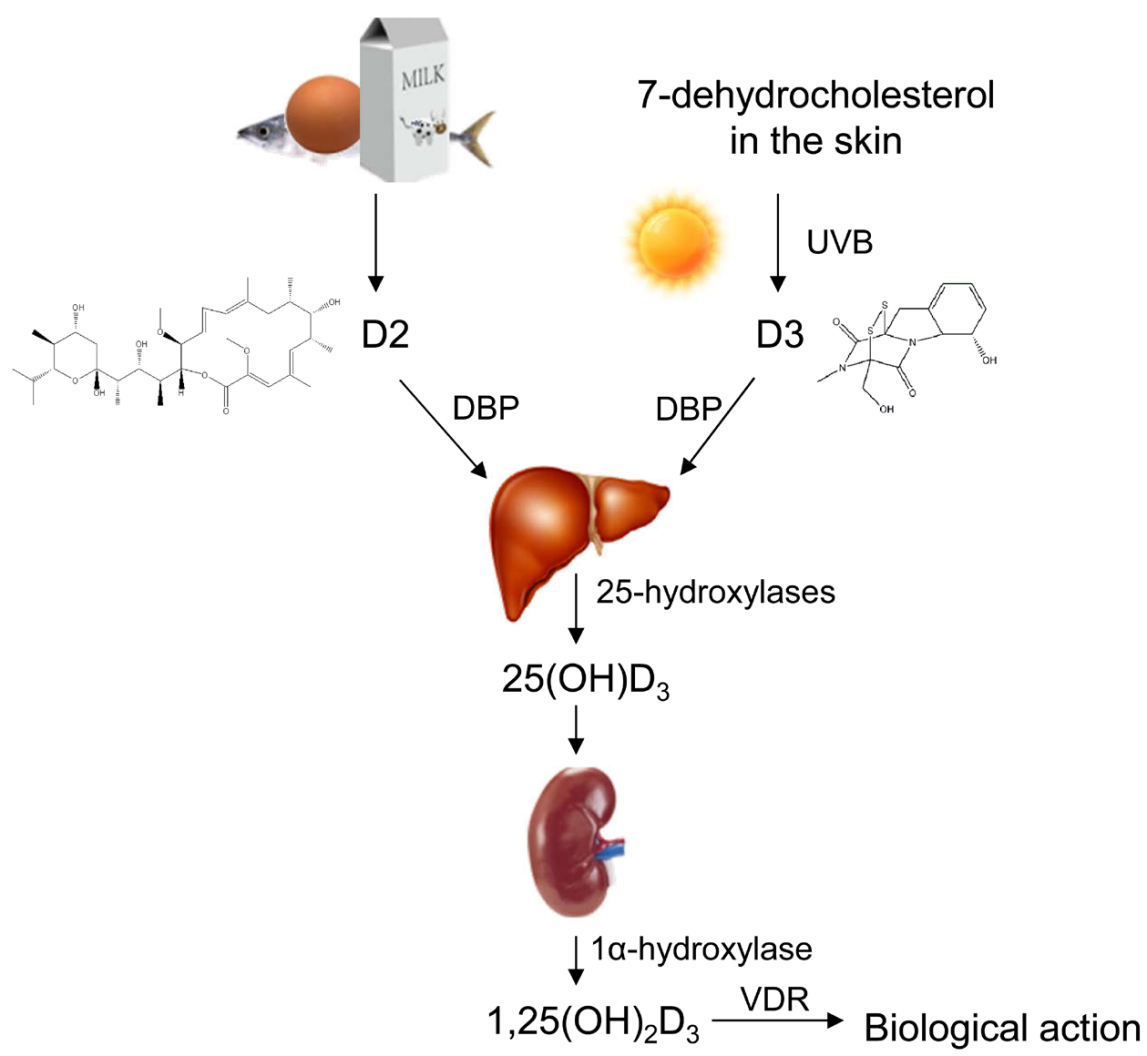
Transforming pathways of vitamin D in human body Vitamin D can be obtained from foods and synthesized through convertion of 7-dehydrocholesterol by UVB in the skin. The absorbed vitamin D transports into liver by binding to vitamin D binding protein (DBP), then vitamin D is hydrolysed into form 25-hydroxyvitamin D_3_ (25(OH)D_3_) by 25-hydroxylase in the liver. Again, 25(OH)D_3_ is hydrolysed into form 1α,25-dihydroxyvitamin D_3_ (1α,25(OH)_2_D_3_) in the kidney.

Since 1α,25-dihydroxyvitamin D (1α,25(OH)_2_D_3_, calcitriol) nor vitamin D is the most active metabolite in our body, vitamin D needs two important cytochrome P450-mediated hydroxylation steps in the metabolism. Firstly, vitamin D obtained from both dietary and skin is converted to 25-hydroxyvitamin D_3_ (25(OH)D_3_) by the hepatic 25-hydroxylases (CYP27A1) after transporting to liver *via* the vitamin D binding protein. Secondly, 25(OH)D_3_ is hydroxylated again in the kidney by the enzyme 25-hydroxyvitamin D-1α-hydroxylase (CYP27B1) to yield calcitriol (Figure 1). Of course, vitamin D metabolism is alternatively proceeded in other organs and/or cells and is regulated by parathyroid hormone, fibroblast growth factor 23, and calcitriol itself [23–26].

### Vitamin D receptor

As mentioned above, the biological function of calcitriol is primarily mediated by vitamin D receptor, which is composed of 427 amino acid residues and belongs to the superfamily of steroid/thyroid hormone receptor [13]. VDR regulates biological function of calcitriol by two mechanisms, one involves transcriptional regulation of nuclear VDR, and the other involves nongenomic signal transduction pathways of membrane VDR [27]. The first one is the most studied anti-tumor mechanism of vitamin D. When activated by calcitriol, the phosphorylated nuclear VDR forms homodimers or heterodimers VDR*-*RXR with one of the retinoid X receptors (RXR), then the calcitriol-VDR*-*RXR complex translocates into nucleus and attaches to the vitamin D response elements (VDREs) in the promoters of target genes, causing the recruitment of co-activators or co-repressors to regulate gene expression in target cells [28, 29]. In addition to the genomic responses mediated by nuclear VDR, membrane VDR mediates rapid responses pathways within 1-2 min to 15-45 min, including intestinal absorption of Ca^2+^ (transcaltachia) [30], secretion of insulin by pancreatic β-cells [31], opening of voltage-gated Ca^2+^ and Cl^−^ channels in osteoblasts and sertoli cells [32, 33], and migration of endothelial cells [34].

## VITAMIN D/VDR AND GASTRIC CANCER: EPIDEMIOLOGICAL DATA

### Ultraviolet B (UVB) and vitamin D in gastric cancer

Abundant vitamin D is synthesized in the skin by UVB, which is commonly regarded as the major vitamin D. Since Frank Garland proposed the UVB-vitamin D-cancer hypothesis in 1980 [7], more and more epidemiology studies support that UVB related vitamin D deficiency is an important risk factor of cancer incidence and mortality in recent years [35–38]. In the stomach, almost all epidemiological data support a strong inverse association between sunlight exposure and cancer incidence and/or mortality rates [34–44] (Table 1).

**Table 1:** Study on correlation of sun exposure and gastric cancer

Study design	Study period /Participants	Vitamin D index	Outcome	Summary of findings	References (year, country)
Ecologic study	1970-1994	TOMS DNA-weighted UVB	Mortality of premature gastric cancer	Inverse correlation *P* < 0.001	Grant et al. (2002, US)
Ecologic study	1961-1990	Average annual hours of solar radiation	Mortality of gastric cancer	Inverse correlation *P* < 0.001	Mizoue (2004, Japan)
Ecologic study	1993-2002	Latitude and Annual erythemally weighted UVB	Mortality and incidence of gastric cancer	Inverse correlation *P* < 0.001	Boscoe et al. (2006, US)
Ecologic study	1990-1994	Latitude and dietary supply factors	Mortality and incidence of gastric cancer	Inverse correlation *P* < 0.05	Grant et al. (2006, western European)
Ecologic study	1970-1994	TOMS DNA-weighted UVB	Mortality of gastric cancer	Inverse correlation *P* < 0.001	Grant et al. (2006, US)
Ecologic study	1978-1992	Latitude, skin cancer and melanoma	Mortality of gastric cancer	Inverse correlation *p* < 0.01	Grant et al. (2007, Spain)
Case-control	416,134 cases 3,776,501controls	Skin cancer and sunexposure	Incidence of gastric cancer	Inverse correlation SIR: 0.65 95%CI:0.45–0.91	Tuohimaa et al. (2007, Five Continents)
Ecologic study	1950-1994	TOMS DNA-weighted UVB	Mortality of gastric cancer	Inverse correlation *p* < 0.001	Grant et al. (2010, US)
Ecologic study	1998–2002	UVB from NASA database and GIS methods	Mortality of gastric cancer	Inverse correlation *p* < 0.001	Chen et al. (2010, China)
Nested case-control	115,016 cases 987,893controls	Skin cancer	Incidence of gastric cancer	No correlation OR:1.00 95%CI:0.85–1.17	Lindelof et al. (2012, Swedish)
Ecologic study	2000-2002	UVB intensity from NASA database and spatial Kriging method	Mortality of gastric cancer	Inverse correlation HR:0.89 95%CI:0.83-0.95	Chen et al. (2013, China)

Ecological studies in predominantly European populations reported higher cancer survival in areas of higher solar UVB irradiation [39], indicating that high vitamin D status can improve gastric cancer survival. Chen [40] also found the 5-year survival proportions for gastric cancer were inversely associated with ambient UVB in the developing countries. In particular, Boscoe [41] extended the analysis of this relationship to include cancer incidence as well as mortality. After studying over three million cancer cases between 1998-2002 in the United States and three million cancer deaths with daily satellite-measured solar UVB levels between 1993-2002, they found an inverse relationship between solar UVB exposure and cancer incidence and mortality for 10 types of cancer, including gastric cancer. Following this initial finding, the protection role of UVB in gastric cancer was continually reported worldwide [42–44]. The people with deficiency of synthesis vitamin D in the skin are also in the high risk and/or mortality of gastric cancer [40–43]. For example, the premature with inadequate doses of solar UVB radiation showed increased gastric cancer mortality in the U.S. [45], the Blacks with low vitamin D levels were found to have higher risk of cancer incidence and mortality especially in digestive system [46]. To further assess whether patients with skin cancer have an altered risk of developing other cancers, a study found that the patients with skin cancer really have a high risk of developing other cancers, and the standardized incidence ratio of gastric cancer in sunny countries was significant lower than in less sunny countries (SIR(S)/ SIR(L) 0.61, 95%CI 0.38-0.9). Moreover, the apparently protective effect of sun exposure against second primary cancer was more pronounced after non-melanoma skin cancers than melanoma [47]. This result was also reported in an ecologic study of cancer mortality rates in Spain [48]. However, another case control study from Sweden [49] showed divergent result, the cases with the diagnosis of basal cell carcinoma in skin had an increased risk of getting another form of cancer, and had no correlation with gastric cancer (OR 1.00, 95%CI 0.85-1.17). One limitation of this study is the exposure factor bias, whereby the study completely neglects the factor of sun exposure to patients, which may lead to this contradiction. More convincingly, Grant [50] found that solar UVB and vitamin D can reduce the risk of gastric cancer using Hill's criteria for causality. But most of these epidemiological results are from ecologic studies, further investigations to directly measure vitamin D status *in vivo* are needed.

### Vitamin D intake and serum vitamin D in the patients with gastric cancer

Although the protective role of vitamin D from solar UVB on gastric cancer is obvious, the relationship between vitamin D intake and serum vitamin D in the patients with gastric cancer is inconsistent. There are about ten cohort studies in all (Table 2). These studies measured serum concentrations of 25(OH)D_3_ as serum vitamin D status, and assessed dietary vitamin D intake by a diet history questionnaire [51, 52], or food frequency questionnaire [53, 54].

**Table 2:** Studies on vitamin D intake and serum vitamin D status in the patients with gastric cancer

Study design	Participants	Exposure to	Methods of measurement	Outcome	Summary of findings	References (year, country)
Case–control (7y)	723 cases 2,024 controls	Vitamin D intake	Diet history	Risk of gastric cancer	Positive correlation OR:1.35 95%CI:1.00-1.83	Vecchia et al. (1994, Italy)
Case-control (10y)	230 cases 547 controls	Vitamin D intake	Food frequency questionnaire	Risk of gastric cancer	No significant correlation OR:1.33 95%CI:0.80-2.21	Pelucchi et al. (2009, Italy)
Prospective cohort(24y)	1,105 people	Serum 25(OH)D	ELISA	Mortality of upper gastrointestinal cancers	No correlation HR:0.97 95%CI:0.88-1.06	Lin et al. (2012, China)
Prospective cohort( 5.25y)	2084 people	Serum 25(OH)D	ELISA	Risk of gastric cancer	No significant correlation HR:1.77 95%CI:1.16– 2.70	Chen et al. (2007, China)
Pooling project	1,065cases 1,066 controls	Serum 25(OH)D	ELISA	Risk of gastric cancer	Inverse correlation OR=0.65（＞100nmol/L） 95%CI:0.26-1.62	Abnet et al. (2010, US, Finland and China)
Prospective cohort(14y)	51,529 men	Predicted 25(OH)D	Model predicting 25(OH)D	Risk of gastric cancer	Inverse correlation RR = 0.58 95%CI=0.26-1.33	Giovannucci et al. ( 2006, US)
Retrospective case-control	49cases 49controls	Serum 25(OH)D	ELISA	Risk of gastric adenocarcinoma with VD deficiency	Positive correlation OR=3.8 95%CI:1.42-10.18	Vyas et al. (2016, US)
Case-control	68 cases 20 controls	Serum 25(OH)D	ELISA	Level in gastric cancer	Increased in gastric cancer P=0.036	Fidan et al. (2010, Turkey)
Observational study	197cases	Serum 25(OH)D	ELISA	An independent prognostic factor of gastric cancer	Inverse correlation P=0.019	Ren et al. (2012, china)
Prospective cohort	43,468White men 481 Black men	Vitamin D Intake	Dietary questionnaire	Gastric cancer mortality and incidence with hypovitaminosis D	Increased incidence RR=1.57 95% CI=1.16-2.11 Increased mortality RR=2.27 95%CI=1.57-3.28	Giovannucci et al. (2006, US)

The latest cohort study from China [55]investigated whether baseline serum 25(OH)D_3_ concentrations were associated with all-cause mortality and cause-specific mortality rates over 24 years of follow-up (1986-2010). They found that serum 25(OH)D_3_ concentrations were not associated with the mortality of upper gastrointestinal cancer in Chinese population. Similarly, Pelucchi [53] and Chen [56] did not find a significant relationship between dietary intake of vitamin D and risk of gastric cancer. However, another study [57] reported a non-statistically significant but suggestive inverse relationship between vitamin D status and risk of gastric cancer. In particular, the Cohort Consortium Vitamin D Pooling Project of Rarer Cancers (VDPP) brought together 10 cohorts to conduct a prospective study of the association between vitamin D status and upper gastrointestinal cancers. In multivariate adjusted models, circulating 25(OH)D_3_ concentrations were not significantly associated with the risk of upper gastrointestinal cancer, but higher concentrations of 25(OH)D ( > 100 nmol/L) were inversely associated with the risk of gastric cancer (OR:0.65, 95% CI 0.26-1.62) [57]. However, some studies found a positive association between vitamin D and the risk of gastric cancer [52, 58]. Such as in a case-control study from Italy [52], a significant positive association was reported between vitamin D intake and the risk of gastric cancer (OR: 1.35, 95% CI: 1.00-1.83).

On the contrary, some studies support the notion of vitamin D-reduced the incidence and mortality of gastric cancer [54–56]. A retrospective case-control study revealed that the prevalence of vitamin D deficiency in gastric adenocarcinoma group was significantly higher than that in the control group (OR: 3.8, 95% CI:1.42-10.18, P:0.0079), suggesting a positive correlation between vitamin D deficiency and incidence of gastric adenocarcinoma [59]. A Health Professionals Follow-Up study found an increment of 25 nmol/L in predicted 25(OH)D_3_ level was associated with a 17% reduction in total cancer incidence, a 29% reduction in total cancer mortality, and a 45% reduction in digestive cancer mortality, particularly in gastric cancer. It was therefore recommended that at least 1500 IU/day may be necessary for the vitamin D supplementation to prevent digestive cancer mortality [60]. Ren [61] also reported an inverse association of serum 25(OH)D_3_ concentrations with clinical stage and lymph node metastasis of gastric cancer, suggesting that serum vitamin D level is a significant independent prognostic factor and vitamin D deficiency is associated with poor prognosis in gastric cancer.

As mentioned earlier, dietary vitamin D intake is a minimum source for the levels of circulating serum vitamin D since only 3 ng/mL differences were found in measured serum vitamin D between high and low dietary intake [62]. Besides, serum vitamin D status in most studies were based on one-time blood collection only, it is obvious that a single measurement of serum vitamin D cannot reflect real exposure to vitamin D in an etiologically relevant period. Even more, vitamin D status not just refer 25(OH)D_3_ but include 1α,25(OH)_2_D_3_ and vitamin D binding to DBP since a very little vitamin D circulates as a free form of 25(OH)D_3_ [63]. All these may make an inconsistent relationship between vitamin D status and gastric cancer.

### Vitamin D receptor in gastric cancer

The biological function of vitamin D, especially its anticancer effects, are largely through activation of VDR [64, 65], which is required to suppress tumorigenesis and may be a new target for cancer chemoprevention and/or chemotherapy [66]. It has been reported that a higher VDR expression is associated with reduced mortality, favorable tumor characteristics and an improved prognosis in breast, prostate and colon cancer [67–70]. One study from China [71] reported similar results that VDR expression was significantly lower in gastric cancer tissues, and that among cancer tissues VDR was higher expressed in well and moderate differentiated tissues and in small tumors, indicating that VDR could be a prognostic factor for gastric cancer.

Most VDR gen polymorphisms were identified since 1997 [72], and about six gen polymorphisms were found to be associated with cancers [73–75]. A case-control study [76] revealed a strong relationship between VDR TaqI(T/T) and the susceptibility of Chinese Han population to gastric cancer. Another study in Chinese Han population [77] revealed the patients of gastric cancer with the ƒ allele (Fƒ+ƒƒ) had higher risk of a poorly differentiated type of gastric cancer. This finding has been reproduced in Uygur [78].

### Vitamin D binding protein in gastric cancer

Vitamin D binding protein (DBP), a key protein in vitamin D metabolism, also mediates the biological function of vitamin D [79]. Several studies [80] have investigated serum DBP levels and DBP polymorphisms in association with cancer risk. Humphries [81] has validated DBP as one of the novel biomarkers of human gastric cancer. Two common coding single nucleotide polymorphisms (SNP) were identified in DBP gene, Glu416Asp (rs7041) and Thr420Lys (rs4588) [82]. Zhou [83]found that DBP Thr420Lys and Glu416Asp polymorphism had significant impact on the risk of developing gastrointestinal cancers in Chinese population. So far, the evidence is too little to confirm the relationship between DBP and gastric cancer, but it sheds some light to further study on DBP in gastric cancer.

## VITAMIN D/VDR AND GASTRIC CANCER: LABORATORIAL RESEARCH

### Vitamin D in animal models of gastric cancer

The anti-tumor effects of vitamin D have been extensively studied in animal models. Since vitamin D has the side effect of hypercalcemia, its analogs are widely used. Hiroki [84] found that 1α(OH)D_3_, a synthetic analogue of vitamin D_3_, markedly inhibited the inductions of ornithine decarboxylase (ODC) activity by promoters of carcinogenesis in the stomach, suggesting an anti-tumor effect of vitamin D on gastric carcinogenesis. Vitamin D analogue 1α(OH)D_3_ largely reduced the incidence of gastrointestinal tumors induced by N-methyl-N’-nitro-N-nitrosoguanidine in male Wistar rats [85]. 24R, 25-dihydroxyvitaminD_3_, a vitamin D_3_ derivative, also had chemopreventive effects on glandular stomach carcinogenesis in rats possibly by influencing calcium pharmacodynamics [86]. All of these results suggest that vitamin D and its analogs can inhibit the occurrence and development of gastric cancer in animal models.

### Vitamin D regulation of specific signaling pathways in gastric cancer cells

**Figure 2:**
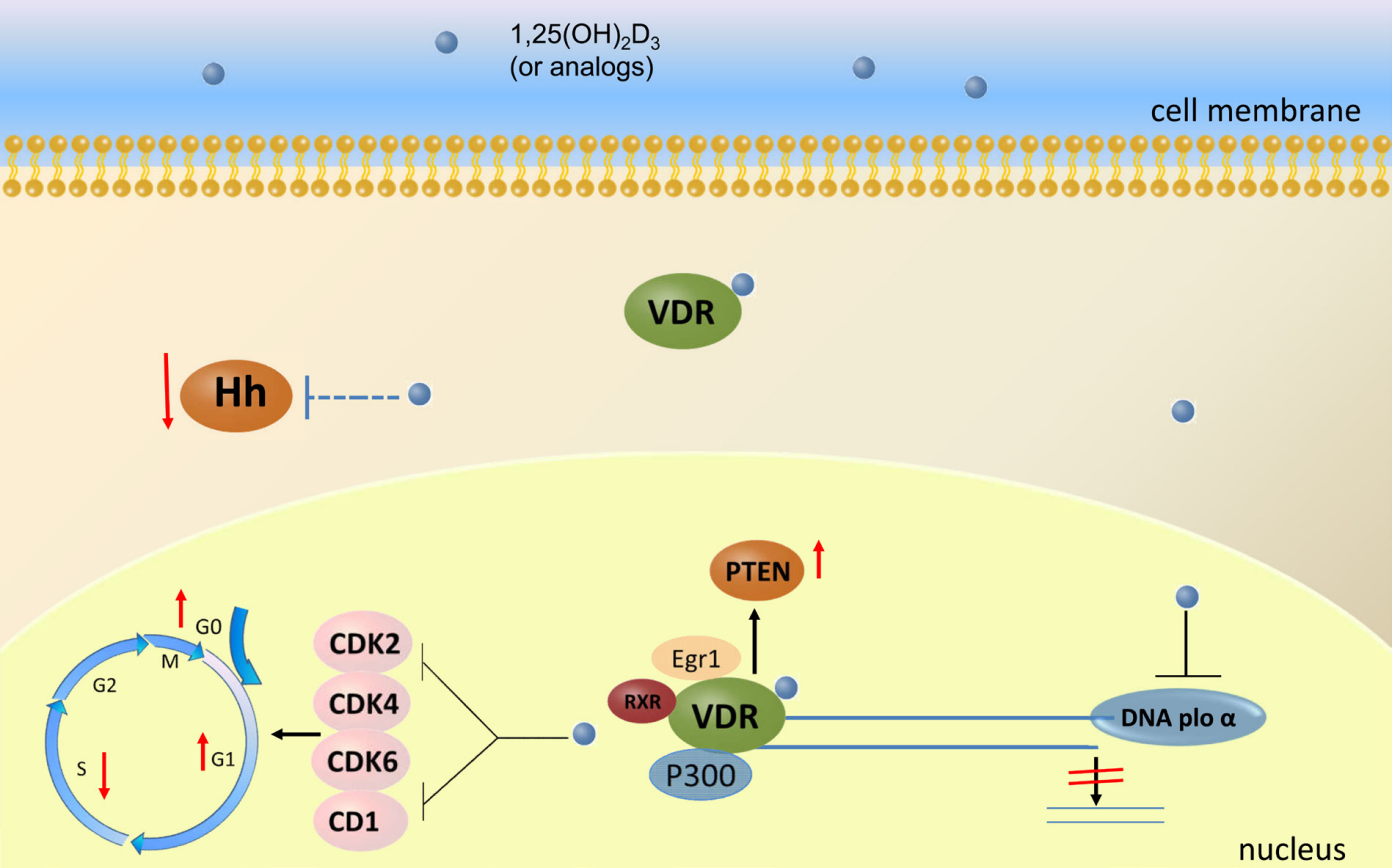
The anti-cancer mechanisms mediated by vitamin D and its analogues through VDR activation in gastric cancer cells Four cellular signaling pathways are likely involved in the anti-cancer mechanisms of vitamin D: 1) it inhibits mammalian DNA polymerase α to halt NUGC-3 human gastric cancer cells at the G1 phase in the cell cycle, 2) it blocks cell cycle of gastric cancer cells by decreasing the expression of cyclin-dependent kinase, CDK2, CDK4, CDK6 and Cyclin D1, 3) together with VDR, Egr-1 and p300 it induces gastric cancer cell apoptosis through PTEN upregulation, and 4) it acts as an antagonist of hedgehog signaling to suppress viability of gastric cancer cells. CDK: cyclin dependent kinase; PTEN: phosphatase and tensin homolog deleted on chromosome 10; Egr-1: early growth response gene 1; Hh: hedgehog.

Although abundant evidences from epidemical studies and animal models suggest vitamin D could obviously inhibit gastric cancer *in vivo*, its antitumor mechanisms are unclear. Some evidence indicate that vitamin D could block cell cycle, induce apoptosis and inhibit cell invasion and metastasis (Figure 2.) [87, 88].

Selective inhibitors of mammalian DNA polymerase α, vitamin D_2_ and D_3_ could halt NUGC-3 human gastric cancer cells at the G1 phase in the cell cycle [89]. Park [90] found that 19-nor-1,25-dihydroxyvitamin D_2_, a vitamin D analog, could block cell cycle of MKN45 gastric cancer cells by decreasing the expression of cyclin-dependent kinase(CDK), CDK2, CDK4, CDK6 and Cyclin D1. Functional VDR elements have been identified in the promoter of PTEN, suggesting that vitamin D may play a role in the regulation of PTEN expression as a nuclear transcription factor [91]. A study [92] demonstrated that vitamin D induced apoptosis through PTEN upregulation in HGC-27 gastric cancer cells, and that vitamin D receptor, Egr-1 and p300 induced PTEN expression in a synergistic fashion. Another study indicated that EB1089, a vitamin D analog induced gastric cancer cells apoptosis through a VDR and mitochondrial apoptosis pathway, which was blocked by treating the cells with VDR siRNA or butin, an inhibitor of the mitochondrial apoptosis pathway [93].

Since Hedgehog signaling pathway plays an important role in the pathogenesis and the prognosis of gastric cancer, targeting this pathway is a new potential therapeutic opportunity in gastric cancer [94]. Vitamin D_3_ may act as an antagonist of hedgehog signaling to suppress viability of gastric cancer cells, and it also has a synergistic effect with other anticancer drugs by reducing mRNA expression of the target genes of hedgehog signaling ( Ptch1, Gli1, cyclin D1 and bcl2) [95]. Co-treatment with cisplatin and 1α,25(OH)_2_D_3_ enhanced cisplatin-mediated cell growth inhibition and cell apoptosis of human gastric cancer cells with an upregulation of Bax, a decrease in ERK and AKT phosphorylation levels, and an increase in p21 and p27 levels [96].

### Vitamin D up-protein1

Vitamin D_3_ upregulated protein 1 (VDUP1) is a 46 kDa protein upregulated by 1α,25(OH)_2_D_3_ [97]. VDUP1 has an antitumor activity by forming a transcriptional repressor complex, which induced cell-cycle arrest at the G0/G1 phase and suppressed cell invasiveness and tumor metastasis [98, 99]. In clinic, VDUP1 expression is significantly lower in gastric cancer tissue than in their adjacent normal tissue and the downregulation of VDUP1 expression is associated with poor prognosis [100]. Kwon [101] found that VDUP1 negatively regulates Helicobacter pylori-associated gastric carcinogenesis in mice by disrupting cell growth and inhibiting the induction of TNFa, NF-kB and COX-2, suggesting that VDUP1 may serve as a potential target for the development of anticancer agents for gastric cancer.

### Viatmin D with H. pylori and microRNA

*Helicobacter pylori (Hp)* infection plays an important pathogenic role in most gastric cancer cases [102, 103]. International Agency for Research on Cancer (IARC) classified *Hp* as a group 1 carcinogen in 1994, and reconfirmed this classification in 2009 [2]. A cross-sectional study found a significant positive correlation between the levels of serum 25-OH vitamin D and serum *Hp* specific IgG antibody titers, indicating that vitamin D analog may have antibacterial action against *Hp* [104]. Kouichi Hosoda [105] further confirmed Vitamin D_3_ decomposition product (VDP1) can exert an antibacterial action against *Hp* by inducing a collapse of cell membrane structures of *Hp* and ultimately lysing the bacterial cells. These findings suggest that VDP1 may become a new antibacterial substance against *Hp*.

MicroRNAs (miRNAs) are short, single strands of noncoding RNA with important functions in mRNA translation and regulation of cell cycle and apoptosis [106]. It was reported that miR145 induced by 1α, 25(OH)_2_D_3_ through VDR could inhibit colony formation, gastric cancer cell viability and induce cell arrest at S-phase by targeting E2F3 and CDK6. This might hold promise for prognosis and therapeutic strategies for gastric cancer [107].

### Vitamin D/VDR in immunity

In the last few years, accumulating evidence indicates an important modulatory role of vitamin D/VDR in adaptive and innate immune cells [108–110], which is distinct from their classical anti-tumor roles. After identification of VDR in series immune cells, numerous cellular and molecular targets of VDR in the immune system have been elaborated [111, 112]. VDR regulates all stages of a T cells life, ranging from development to differentiation and elicitation of effector functions [113]. Likewise, VDR is essential for Th2 cell function, and vitamin D could increase the activities of regulatory T cells and Th2 cells while suppressing Th1 cell activity [114, 115]. In antigen-presenting cells, vitamin D is believed to program dendritic cells (DC) for tolerance, dampen their ability to activate effector T-cell generation, and enhance their potential to induce anti-inflammatory regulatory T (Treg) cells. Vitamin D also interacts with DC to influence their migration and their capacity to instruct T cells and hence to initiate, fine tune or dampen immune reactions [116]. On the other hand, vitamin D-treated DCs are significantly more potent in driving differentiation of IL-22-producing T cells and are markedly enhanced to secrete TNF-α, IL-6, IL-1β and IL-23 [117]. However, despite compelling evidence for the roles of vitamin D/VDR in immunity, there are no studies on vitamin D/VDR in gastric cancer immunity.

### Vitamin D and gastric cancer: clinical trial

To date, it is scarce for the clinical trial directly exploring vitamin D to potentially treat gastric cancer [118, 119]. In a 4 year, population-based, double-blind, randomized placebo-controlled trial [119], Joan et al. found that the relative risk of developing cancer was 0.232 for the calcium plus vitamin D group and 0.587 for the calcium alone group, and that serum 25-hydroxyvitamin D concentrations were significant, independent predictors of cancer risk. As *Hp* is regarded as an independent risk factor of gastric cancer, Kawaura et al. [118] tested whether long term 1α(OH)D_3_ administration could inhibit *Hp* infection, and they found that *Hp* infection rate was significantly lower in subjects with 1α(OH)D_3_ treatment than those without treatment. This study reconfirms vitamin D analog has antibacterial action against *Hp*.

## CONCLUSIONS

Vitamin D has received extensive attention in recent years, especially after Mark [120] found vitamin D could promote protein homeostasis and longevity in nematodes. Most current evidence suggests that vitamin D is inversely associated with the morbidity and mortality of gastric cancer. Not only laboratorial studies at the levels of cells, tissues and animal models but also clinical trial support an anti-cancer role of vitamin D. However, the epidemiological data are still paradoxical. The studies utilizing ultraviolet B exposure as a main measurement consistently show the increased risk of gastric cancer with vitamin D deficiency, but some studies measuring serum 25(OH)D_3_ levels in human body do not support this notion. As discussed above, imprecise and inconsecutive assessment of serum vitamin D status may lead to the obscure relationship between 25(OH)D_3_ levels and gastric cancer risk. The laboratorial studies demonstrate that vitamin D and its metabolites activate VDR to inhibit viability, proliferation and metastasis of gastric cancer cells, and also explore the underlying molecular mechanisms against gastric tumorigenesis and progression. Furthermore, vitamin D metabolites or analogues might also inhibit *Hp* infection and *Hp*-associated gastric cancer. Although basic research supports the protective effects of vitamin D against gastric cancer, further studies are needed to elucidate its anti-tumour mechanisms, especial its interaction with VDR. At last but not least, large-scale and long-term clinical randomized controlled trials (RCTs) are necessary to make a definite conclusion whether vitamin D can really offer preventive and/or therapeutic benefits to gastric cancer.

## Abbreviations

VDR: vitamin D receptor
1α,25(OH)_2_D_3_: 1α,25-dihydroxyvitamin D
25(OH)D_3_: 25-hydroxyvitamin D
UVB: Ultraviolet B
DBP: Vitamin D binding protein
VDUP1: Vitamin D3 upregulated protein 1
PTEN: phosphatase and tensin homolog deleted on chromosome 10
Hp: Helicobacter pylori
DC: dendritic cell.
